# Nanodissected elastically loaded clathrin lattices relax to increased curvature

**DOI:** 10.1126/sciadv.abg9934

**Published:** 2021-08-13

**Authors:** Grigory Tagiltsev, Christoph A. Haselwandter, Simon Scheuring

**Affiliations:** 1 Department of Anesthesiology, Weill Cornell Medicine, 1300 York Avenue, New York, NY 10065, USA.; 2 Department of Physiology and Biophysics, Weill Cornell Medicine, 1300 York Avenue, New York, NY 10065, USA.; 3 Department of Physics and Astronomy, University of Southern California, Los Angeles, CA 90089, USA.; 4 Department of Quantitative and Computational Biology, University of Southern California, Los Angeles, CA 90089, USA.

## Abstract

Clathrin-mediated endocytosis (CME) is the major endocytosis pathway for the specific internalization of large compounds, growth factors, and receptors. Formation of internalized vesicles from the flat plasma membrane is accompanied by maturation of cytoplasmic clathrin coats. How clathrin coats mature and the mechanistic role of clathrin coats are still largely unknown. Maturation models proposed clathrin coats to mature at constant radius or constant area, driven by molecular actions or elastic energy. Here, combining high-speed atomic force microscopy (HS-AFM) imaging, HS-AFM nanodissection, and elasticity theory, we show that clathrin lattices deviating from the intrinsic curvature of clathrin form elastically loaded assemblies. Upon nanodissection of the clathrin network, the stored elastic energy in these lattices drives lattice relaxation to accommodate an ideal area-curvature ratio toward the formation of closed clathrin-coated vesicles. Our work supports that the release of elastic energy stored in curvature-frustrated clathrin lattices could play a major role in CME.

## Teaser

Clathrin-coated pits mature stochastically through release of elastic energy stored in the clathrin coat.

## INTRODUCTION

Clathrin-mediated endocytosis (CME) is one of the key transmembrane traffic pathways in eukaryotes. It is involved in major physiological processes such as internalization of growth factors and receptors (*1*) and the uptake of macromolecules (*2*). CME proceeds through the formation of a clathrin-covered plasma membrane region that gradually buds out as a clathrin-coated pit (CCP) and is finally released into the cytoplasm as a clathrin-coated vesicle (CCV). CME has been studied extensively over the past 50 years (*3*–*5*): In brief, depending on the biological process, cargo molecules can trigger CME by binding to corresponding transmembrane receptors from the extracellular side. Next, the cargo-bound receptors recruit various adaptor proteins (e.g., AP2, AP180/CALM, and epsins) on the intracellular side. Then, the adaptor proteins recruit clathrin that polymerizes into a lattice and forms a coat around a vesicle that originates from the budding area and is finally severed from the plasma membrane through the action of dynamin. The building block of clathrin coats is the clathrin triskelion, a trimeric pyramid, with extended curved and flexible arms (*6*).

Our view of CME has been shaped by early carbon-platinum replica electron microscopy (EM) studies (*7*): These three-dimensional (3D) images depicted clathrin lattices of varying size and morphology, comprising large flat lattices composed of hexagonal clathrin assemblies, and curved lattices comprising pentagons. Topologically, hexagonal lattices cannot form closed convex polyhedra such as CCVs. Therefore, pentagonal insertions are required to form curved and closed 3D structures. Knowing that the final state is a spherical CCV, with an architecture resembling a soccer ball comprising hexagons and pentagons (*6*, *7*), researchers put intuitive order into the EM snapshots, where the flat hexagonal lattices represent the earliest and the more curved CCPs that comprise increased numbers of pentagons, the later stages of CME. These observations suggested that first a flat clathrin lattice is formed via polymerization, which is then internally remodeled to produce CCP curvature. In this “constant area model” of CME, the area of the clathrin lattice is assumed to be constant during CCP curvature formation, with the transition from flat clathrin lattices to curved CCPs being driven by changes in the structure of clathrin lattices (fig. S1A).

The seemingly obvious reasoning underlying the constant area model of CME has been challenged by detailed geometrical considerations (*8*) and structural analysis of reconstituted clathrin cages (*6*): To introduce a pentagon into a flat hexagonal clathrin lattice via clathrin triskelion removal, the clathrin lattice would have to transition through a series of large-scale rearrangements, for which experimental evidence has been lacking (*8*). In addition, cryo-EM structures of clathrin cages (*6*, *9*) highlighted extended and intricate interactions of clathrin triskelia with their neighbors in clathrin lattices, suggesting that spontaneous removal of even a single triskelion in a clathrin lattice would be highly unlikely (*8*). These considerations suggested the “constant curvature model” of CME, in which CCVs assemble through the growth of curved clathrin lattices comprising pentagons, with the radius of curvature of the clathrin lattice taking a constant value during CCV formation (fig. S1B).

The constant area and constant curvature models of CME both have appealing features but do not capture all aspects of CME. While it was reported that CCPs contain traces of the clathrin coat disassembly machinery (*10*), which could mediate clathrin coat topology changes via partial dis- and re-assembly of the clathrin lattice (*11*), the constant area model would call for large energy consumption and an extended time to transition from flat to curved clathrin lattices (fig. S1A). In contrast, the constant curvature model implies that clathrin triskelia readily assemble into curved structures comprising pentagons, with lattice growth progressively accompanying CCV formation. This model is, however, at odds with a large body of EM data showing, besides highly curved clathrin assemblies, CCPs of various morphologies including extended, flat hexagonal clathrin lattices and CCP intermediates with small curvatures that are incompatible with the direct formation of highly curved CCV-like CCPs (fig. S1B) (*7*). The constant area and constant curvature models of CME make distinct assumptions regarding the temporal evolution of CCP area and curvature during CME events (fig. S1, A and B, right), but correlative fluorescence and EM studies have so far not been able to produce a consensus about the clathrin coat maturation mechanism (*12*, *13*). It has been proposed that both constant area and constant curvature models may apply (*12*). However, the constant curvature model formally rejects clathrin polygon interconversions, which are a key component of the constant area model. Last, live-cell fluorescence imaging reported nonterminal, failed CME events (*14*), which need to be accounted for in a coherent model of CME.

The constant area and constant curvature models of CME suggest that further progress on the clathrin coat maturation mechanism requires quantitative measurements of the CCP area and radius of curvature. Here, we used high-speed atomic force microscopy (HS-AFM) (*15*, *16*) to investigate clathrin coats on native plasma membranes following cell unroofing. HS-AFM has proven to be a powerful tool for the study of other membrane trafficking systems such as annexin V (*17*), Endosomal Sorting Complex Required for Transport III (ESCRT-III) (*18*–*20*), and dynamin (*21*, *22*). Providing images with precise real-space 3D information, HS-AFM allows us to correlate clathrin architecture with CCP area and radius of curvature. Interpreting our data within the framework of elasticity theory, we propose a clathrin coat maturation model centered around the CCP energy landscape. Elastic energy stored in the clathrin coat has previously been proposed to play a role in CCP maturation (*7*, *23*). While the constant area and constant curvature models of CME postulate a fixed sequence of CME events, the CCP energy landscape model provides a complementary, nonprocessive description of CCP maturation that is agnostic about the precise sequence of CME events. As a result, this elastic model of CCP maturation does not require detailed assumptions about the temporal evolution of CCP area and curvature during CME events, which is difficult to observe experimentally. According to the CCP energy landscape model, clathrin lattices with curvatures less than the intrinsic curvature of clathrin triskelia are elastically loaded. This stored elastic energy drives lattice bending into highly curved CCPs. We test and validate this CCP energy landscape model using the HS-AFM tip as a nanoscalpel: Induction of local mechanical perturbations to the clathrin lattice leads to spontaneous lattice relaxation toward states with increased curvature.

## RESULTS

### 3D morphology analysis does not support current CCP maturation models

As detailed above, the two major clathrin coat maturation models make narrow predictions about the clathrin coat sizes and/or curvatures that should be found among CCPs on cell membranes (fig. S1). As reasoned in previous studies (*12*, *13*), a statistical set of observations upon cell unroofing, halting CME at any instant, should be representative of the process and comparable to the models’ predictions. Here, we used HS-AFM to analyze CCPs on unroofed PTK2 (*Potorous tridactylis* epithelial kidney) cell membranes. HS-AFM provides real-space 3D data with nanometer lateral and vertical resolution in liquid at ambient temperature and pressure. The PTK2 cell line has previously been exploited to study CME using EM and fluorescence microscopy (*24*, *25*): In PTK2 cells, comparable to other cell lines, CME occurs in ~3 min and results in CCVs of ~55-nm radius. CCPs on unroofed PTK2 cell membranes did not evolve in time, even when imaged for tens of minutes, while CME typically completes in a few minutes (*24*–*26*). Hence, the observed CCPs and their characteristics represent stalled intermediates that can be considered to be in static equilibrium.

**Fig. 1:**
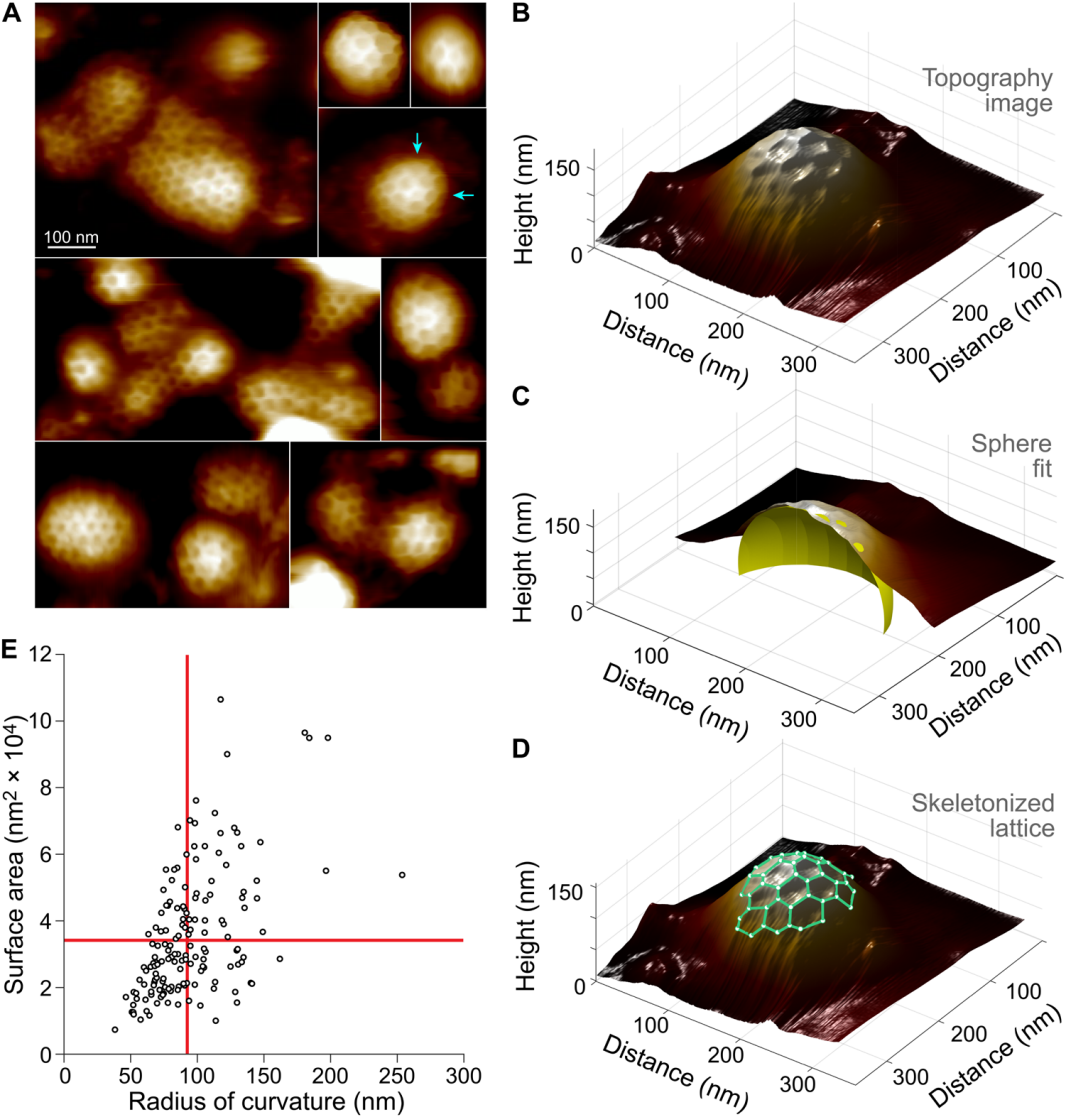
HS-AFM 3D morphology analysis of CCPs on plasma membranes of freshly unroofed PTK2 cells. (**A**) CCPs of various sizes and morphologies observed on plasma membranes of freshly unroofed PTK2 cells (arrows indicate examples of pentagonal insertions). (**B**) 3D representation of an individual CCP. (**C**) Spherical sector (yellow spherical cap) fit of an individual CCP. The fit determines the CCP radius of curvature and surface area (above 0-height level defined by the background height of the surrounding membrane). (**D**) Partial skeleton of clathrin lattice (green lines). The lattice skeleton describes the conformation of each clathrin triskelion in 3D space. (**E**) Distribution analysis of *n* = 128 CCPs visualized by HS-AFM according to their surface area versus radius of curvature. The observed distribution of the CCP area and radius of curvature does not follow the predictions of the constant area (horizontal red line) and constant curvature (vertical red line) models obtained from the mean CCP surface area and radius of curvature, respectively (see fig. S1).

We found CCPs of various sizes, shapes, and protrusion heights (Fig. 1A). From the HS-AFM 3D data (Fig. 1B), the CCP surface area and radius of curvature were determined through fits to spherical caps (Fig. 1C). While the HS-AFM cannot image the CCPs from the side and therefore cannot directly measure the angle θ between the (flat) plasma membrane and the invagination at the foot of the CCP (*13*), θ can be estimated from the fitted spherical caps. Furthermore, the detected clathrin lattices on the CCPs were 3D skeletonized, revealing the polygonal clathrin lattice architecture on the surface of curved CCPs (Fig. 1D). With surface area, curvature, and clathrin lattice architecture in hand for each CCP, we first asked whether the constant area (fig. S1A) or the constant curvature (fig. S1B) model better accounted for the data. Plotting CCP surface area versus CCP radius of curvature for all observed CCPs, we found that neither the constant area nor the constant curvature model favorably compared with the data (Fig. 1E). The data distributed roughly diagonally in an area versus radius of curvature plot (and in between the predictions of the two models), suggesting that CCP area increases with CCP radius of curvature. Given that both models did not satisfactorily describe the observed CCP shapes, we decided that a deeper analysis was necessary, with the aim to propose a novel, and more inclusive, model for CCP maturation.

### Clathrin triskelia adopt various distorted conformations in CCPs

**Fig. 2:**
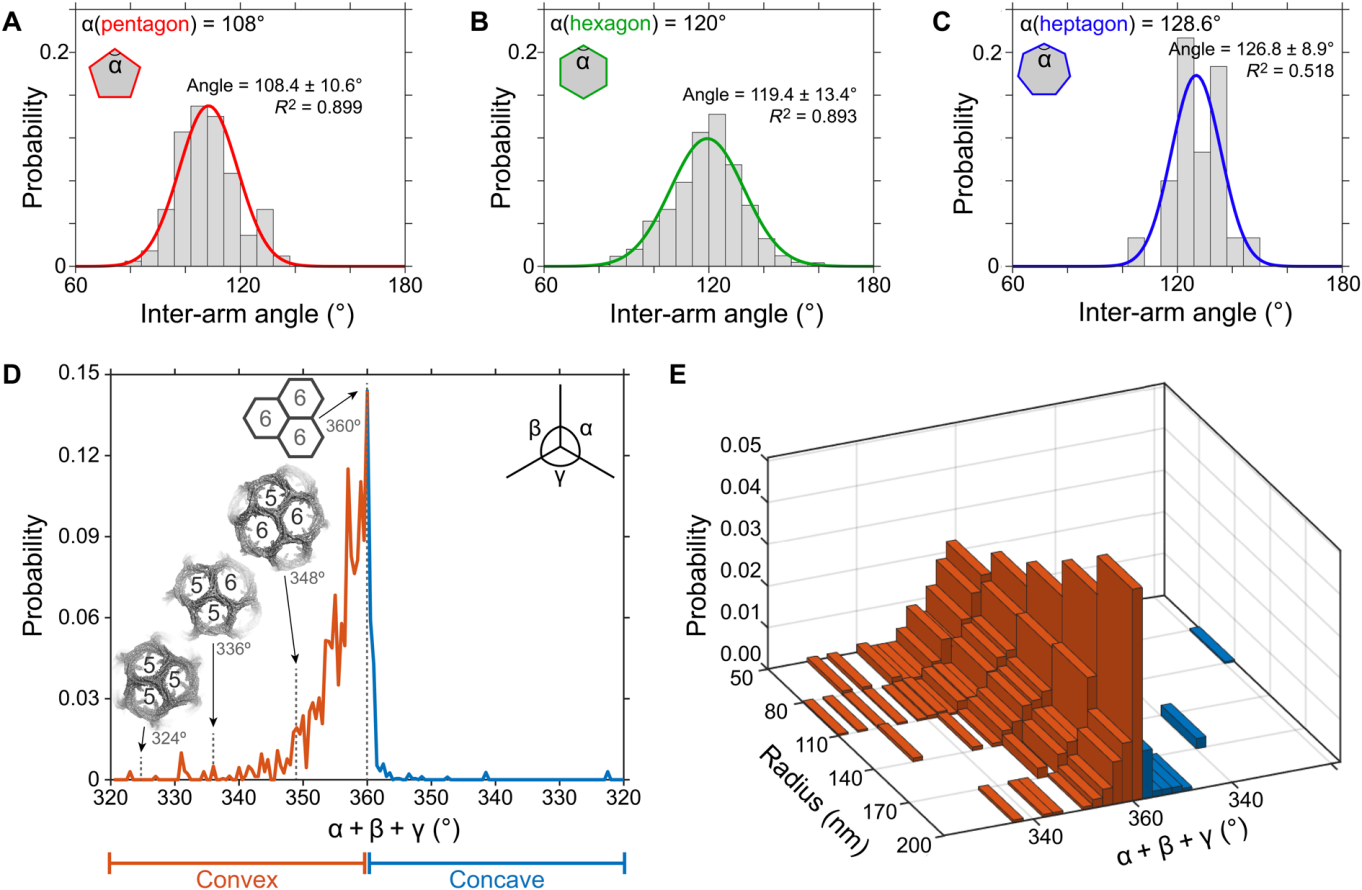
Clathrin inter-arm angles and triskelia conformations in CCPs in 3D space and their correlation with the CCP global curvature. Distributions of inter-arm angles (gray bins) of (**A**) pentagons, (**B**) hexagons, and (**C**) heptagons. The fitted normal distributions of pentagons [red line in (A)], hexagons [green line in (B)], and heptagons [blue line in (C)] peak at mean values approximately equal to the internal angles of the corresponding regular polygons. Fitting statistics are shown in the graphs. (**D**) Distribution of the sum of the three inter-arm angles in triskelia (α + β + γ). Clathrin triskelia conformations were mainly convex (α + β + γ < 360°, red trace) or flat (α + β + γ = 360°), with a residual number of concave (α + β + γ > 360°, blue trace) conformations. The observed convex triskelia do not display the inter-arm angle sums associated with assemblies of regular polygons [insets: (*9*)]. (**E**) Correlation of CCP radius of curvature and inter-arm angle sum in triskelia. CCPs with smaller radius of curvature tend to have more convex clathrin triskelia.

The HS-AFM topographic data combined with sphere fitting and lattice skeletonizing (Fig. 1, B to D) allowed us to extract lattice parameters in irregular CCPs and search for geometric rules—if there were any. Because CCP architecture directly depends on the conformation of each of its constituent clathrin triskelia, we assessed the statistics of clathrin inter-arm angles in pentagons, hexagons, and heptagons (Fig. 2, A to C). Let us first recall some basic geometric properties of regular (flat) polygons: the inter-arm angles are 108° in a regular pentagon, 120° in a regular hexagon, and 128.6° in a regular heptagon; the sum of the three inter-arm angles of a flat triskelion is 360°. In contrast, for triskelia with arms that bend away from the plane to adopt a pyramidal conformation, the sum of the three inter-arm angles is <360° (fig. S2).

Plotting the clathrin inter-arm angles for pentagons, hexagons, and heptagons (Fig. 2, A to C), we found normal distributions around the angles 108.4°, 119.4°, and 126.8°, respectively, close to the corresponding values 108°, 120°, and 128.6° for regular polygons. Thus, all three polygons tend to be regular on average. However, the angular distributions are wide (±~10°), especially for the inter-arm angles in hexagons, which should be a paradigm for flat tiling (Fig. 2B). Thus, while substantial deviations from a regular polygonal geometry are detected, overall CCP curvature must be achieved through the assembly of different polygon types. We therefore calculated the sum of the inter-arm angles at each vertex (i.e., triskelia hub) in the observed CCPs and plotted its probability distribution (Fig. 2D). The sum of the inter-arm angles did not distribute around the values 348°, 336°, or 324° expected for triskelia participating in the polygonal configurations 6-6-5, 6-5-5, or 5-5-5, where 5 and 6 denote the regular pentagon and the regular hexagon, respectively (Fig. 2D, insets) (*9*). In contrast, triskelia adopt a wide range of conformations, with inter-arm angle sums <360° indicative of pyramidal triskelia conformations. This wide distribution of the sum of the inter-arm angles (in contrast to the sharply defined sums of inter-arm angles expected for configurations of regular polygons: 360°, 348°, 336°, and 324°) (Fig. 2D) is in agreement with the wide distribution of inter-arm angles (Fig. 2, A to C). Binning of the clathrin inter-arm angle sums as a function of the CCP curvature corroborated that more highly curved CCPs contained more triskelia deviating from the flat lattice geometry, i.e., angle sums <360°, while CCPs with larger radii of curvature contained more flat triskelia, i.e., angle sums ≈360° (Fig. 2E).

From the above results, we concluded that triskelia tend to form regular polygons and that CCP curvature was mainly achieved through insertion of pentagons and bending of the clathrin lattice along the polygon boundaries. Our results imply that, depending on the CCP radius of curvature, triskelia are subjected to substantial distortions, which must be energetically unfavorable. Furthermore, many triskelia in CCPs participate in three neighboring hexagons (6-6-6), rather than in the configurations 6-6-5 and 6-5-5 found for clathrin cages (table S1) (*9*), which is expected to further constrain clathrin lattices in CCPs to energetically unfavorable states deviating strongly from the intrinsic curvature of triskelia (fig. S2).

### The CCP energy landscape

From the above-described analyses, we concluded that the currently proposed models of CME needed amendment, that the CCP area and radius of curvature were both strongly variable, and that triskelia in CCPs were distorted. What mechanistic principles govern CCP maturation toward CCVs?

It was previously proposed that elastic energy stored in the clathrin coats contributed to CCP maturation (*7*, *23*) and that the energetics of clathrin cages could be described by a minimal physical model comprising the favorable triskelion leg interaction energy and the energy needed to distort triskelia away from their preferred shape (*27*). We extended this physical model to CCPs and considered four key contributions to the total energy of a CCP (*E*_CCP_): bending of the plasma membrane (*E*_memb-bend_), lateral tension in the plasma membrane (*E*_memb-tension_), bending of the clathrin coat (*E*_clath-bend_), and clathrin polymerization (*E*_clath-poly_)

$$E_{\text{CCP}}=E_{\text{memb-bend}}+E_{\text{memb-tension}}+E_{\text{clath-bend}}+E_{\text{clath-poly}}$$ (1)

Our HS-AFM experiments show that CCPs take the approximate shape of spherical caps (Fig. 1). We therefore assume a spherical cap geometry of CCPs when calculating *E*_CCP_ in Eq. 1.

**Fig. 3:**
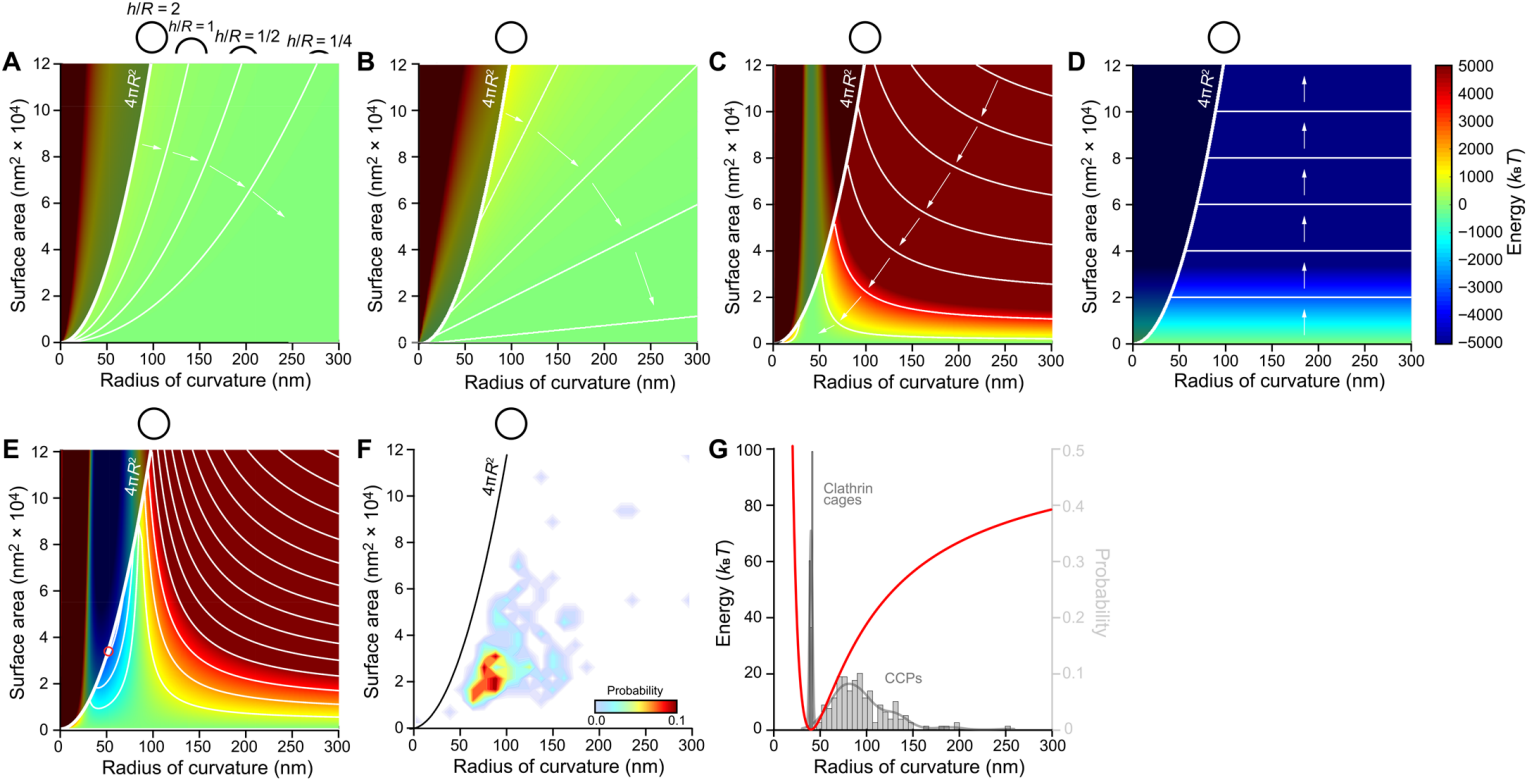
Predicted CCP energy landscape and comparison to experimental data. (**A** to **E**) Heatmaps showing the CCP energy landscape as a function of CCP area and radius of curvature: (A) membrane bending energy in Eq. 2, (B) membrane tension energy in Eq. 3, (C) clathrin coat bending energy in Eq. 4, and (D) clathrin polymerization energy in Eq. 6; (E) combined CCP energy landscape in Eq. 7. The bold curves and black circles in (A) to (F) indicate the surface area–radius of curvature ratio of spheres, following *A* = 4πR^2^; CCPs can only populate the energy landscapes to the right sides of these curves (plot regions on the left sides of *A =* 4π*R*^2^ are shaded dark gray). The thin white curves in (A) to (E) denote equal-energy contours. For (A), equal-energy contours correspond to fixed ratios *h*/*R*, as indicated schematically. Arrows in (A) to (D) indicate the directions in which the respective contributions to the CCP energy landscape drive CCP maturation. The red bold circle in (E) indicates the lowest-energy CCP state with A ≤ 4π*R*^2^, which lies on the *A =* 4π*R*^2^ curve. (**F**) Probability density plot of CCP observations: The highest probability region approximately corresponds to the low-energy CCP states predicted by the combined CCP energy landscape. (**G**) Clathrin bending energy per clathrin arm-arm interaction as a function of the CCP radius of curvature (red line). Clathrin cages, probability distribution of clathrin cages by Morris *et al.* (*9*); CCPs, probability distribution of CCP radius of curvature reported here.

Membrane elasticity theory (*28*, *29*) implies that the energy cost associated with bending the plasma membrane out of its unperturbed configuration is given by $E_{\text{memb-bend}}=\frac{A\kappa_{\text{memb}}}{2}{\left(\frac{2}{R}-\frac{2}{R_{\text{memb}}}\right)}^{2}$, where *A* is the CCP area, κ_memb_ is the membrane bending rigidity, *R* is the CCP radius of curvature, and *R*_memb_ is the intrinsic curvature of the membrane. The value of *R*_memb_ may, for instance, depend on the membrane composition (*30*). We assume here that the energetically preferred state of the membrane is flat, i.e., *R*_memb_ → ∞ (variations of *R*_memb_ away from flatness are explored in fig. S3E). Thus, we have (Fig. 3A)

$$E_{\text{memb-bend}}=\frac{A\kappa_{\text{memb}}}{2}{\left(\frac{2}{R}\right)}^{2}$$ (2)

Only CCP states with *A ≤* 4π*R*^2^, i.e., spherical caps or spheres, are physically accessible. The values of *A* and *R* are experimentally determined here for each CCP (Fig. 1E) and the membrane bending rigidity κ_memb_ ≈ 15 *k*_B_*T* (*29*, *31*). An interesting aspect of Eq. 2 is that the bending energy cost for the formation of a closed vesicle with *A =* 4π*R*^2^ is constant, meaning size independent: *E*_clath-poly_ *= 8*πκ_memb_ ≈ 376 *k*_B_*T*. Thus, all states along the bold curve in Fig. 3A are equal in energy, ≈376 *k*_B_*T*, which is the amount of energy the membrane bending machinery must provide to bend the plasma membrane into a spherical CCP. Similarly, Eq. 2 implies that formation of all CCPs of fixed aspect ratio *h/R* requires an identical amount of membrane bending energy ∝(*h/R*) (Fig. 3A, thin curves).

The work required to form a CCP against membrane tension is given by *E*_memb-tension_ = γΔ*A* (*28*, *32*), where γ is the lateral tension in the plasma membrane and $\Delta A=\frac{A^{2}}{4\pi R^{2}}$ (see derivation in note S2) is the decrease in in-plane membrane area associated with deforming the plasma membrane out of its planar configuration and into the CCP shape. We thus have (Fig. 3B)

$$E_{\text{memb-tension}}=\gamma \frac{A^{2}}{4\pi R^{2}}$$ (3)

The membrane tension γ can take a wide range of values ≈10^−4^ to 1 *k*_B_*T*/nm^2^ in cells. We used an intermediate membrane tension value γ = 0.01 *k*_B_*T*/nm^2^ for all calculations described in the main text, but also considered how changes in the value of γ modify the CCP energy landscape (figs. S3C and S4C and note S4). Note that, unlike *E*_memb-bend_, *E*_memb-tension_ is not constant with the size of spherical CCPs and the CCP aspect ratio but, rather, *E*_memb-tension_ ∝ *h*^2^ and *E*_memb-tension_ is constant for all CCPs with the same ratio *A*/*R* (Fig. 3B, thin lines). Membrane tension was previously observed to affect CCV formation (note S4) (*33*).

Purified clathrin (alone and in the presence of adaptor proteins) forms closed cages in solution with a characteristic radius of ~40 nm (*6*, *9*, *34*). Elastic bending deformations of the clathrin lattice away from its preferred radius of curvature are expected to incur an energetic cost that, in analogy to Eq. 2, is captured by (Fig. 3C) (*35*, *36*)

$$E_{\text{clath-bend}}=\frac{A\kappa_{\text{clath}}}{2}\;{\left(\frac{2}{R}-\frac{2}{R_{\text{clath}}}\right)}^{2}$$ (4)

where κ_clath_ is the bending rigidity of the clathrin lattice and *R*_clath_ is the intrinsic curvature of clathrin triskelia. We assume that the preferred radius of curvature of clathrin triskelia in CCPs takes a similar magnitude as the observed characteristic radius of clathrin cages, and set *R*_clath_ ≈ 40 nm (*6*, *9*, *34*). Equation 4 resembles Eq. 2, used to calculate *E*_memb-bend_. However, in Eq. 2, the intrinsic membrane radius of curvature *R*_memb_ → ∞ (flat); thus, the second term in the brackets is equal to zero for the membrane. In Eq. 4, the sum inside the bracket is equal to zero when *R* = *R*_clath_, yielding a unique minimum of *E*_clath-bend_ at *R* = *R*_clath_ (Fig. 3C). This minimum in *E*_clath-bend_ can only be attained physically for CCPs with area $A\leq 4\pi R_{\text{clath}}^{2}$. To estimate κ_clath_ experimentally, we reconstituted clathrin cages from purified clathrin and measured their mechanical properties using AFM elasticity measurements. We found a cage stiffness *k* ≈ 0.08 N/m, from which we estimated, using thin shell theory (*37*, *38*), a bending rigidity κ_clath_ ≈ 373 *k*_B_*T* (note S1). Various studies have aimed to determine κ_clath_, yielding a wide range of results (*11*, *27*, *33*, *39*)—it was even proposed that κ_clath_ and κ_memb_ are of comparable magnitude (*39*), despite it being fairly counterintuitive that a protein shell should be similarly compliant as a lipid membrane. Computational studies used κ_clath_ ≈ 200 *k*_B_*T* and κ_clath_ ≈ 600 *k*_B_*T* to model clathrin-induced membrane budding (*35*, *36*). In agreement with our determination κ_clath_ > κ_memb_, it was shown that clathrin polymerization alone was able to generate buds out of giant unilamellar vesicles (*40*). The thin curves in Fig. 3C represent clathrin structures with equal *E*_clath-bend_ and highlight how clathrin bending drives CCPs toward highly curved structures with *R* = *R*_clath_ and $A\leq 4\pi R_{\text{clath}}^{2}$.

Clathrin polymerization occurs spontaneously alone or in the presence of adaptor proteins and is likely due to the extensive protein-protein interfaces found in clathrin lattices (*6*, *9*, *34*). Thus, clathrin polymerization is energetically favorable. The clathrin polymerization energy can be estimated from the product of the polymerization energy per clathrin arm-arm interaction *a* and the number of polymerized clathrin arms *N*_poly-arms_, i.e., the total number of clathrin arms, *N*_total_, minus the number of nonpolymerized, open arms *N*_open-arms_

$$E_{\text{clath-poly}}=-{\mathrm{aN}}_{\text{poly-arms}}=-a(N_{\text{total}}-N_{\text{open-arms}})$$ (5)

As we show in note S3, the second term inside the bracket in Eq. 5, characterizing boundary effects due to open clathrin arms, can be neglected for the scenarios of interest here, and the clathrin polymerization energy can thus be written in the form (Fig. 3D)

$$E_{\text{clath-poly}}=-a\frac{A}{A_{\text{clath}}}$$ (6)

where *A*_clath_ ~ 218 nm^2^ is the area sustained by one arm-arm interaction (a third of the clathrin lattice unit cell) (fig. S2E). Direct measurement of *a* in Eq. 6 would require quantitative manipulation of individual clathrin arms in CCPs, which poses technical difficulties. However, we can make a rough estimate of the clathrin polymerization energy by noting that the observed CCPs are stable over extended periods of time, although clathrin is displaced from its energetically preferred configuration. The clathrin lattices in the observed CCPs store an elastic bending energy given by Eq. 4, which imposes an *R*-dependent energy cost per clathrin arm (Fig. 3G, red line). From our CCP radius of curvature probability distribution (Fig. 3G, light gray), we find that the mean CCP radius of curvature is given by *R*_mean_ = 92 nm. Assuming that, for *R* = *R*_mean_, the clathrin bending and clathrin polymerization energies balance each other so that clathrin lattices can grow by sequential clathrin polymerization (*41*) from an approximately flat membrane geometry, one finds *a* ~ 30 *k*_B_*T*. In practice, the observed clathrin lattices in CCPs show defects that may locally permit large curvature deformations with small or no energy penalty, suggesting that the aforementioned estimate of *a* corresponds to an upper bound on *a*. Our estimated value of *a* is in reasonably good agreement with previous experimental and theoretical estimates of *a*: Values between *a* ~ 15 *k*_B_*T* and *a* ~ 28 *k*_B_*T* were estimated from models of the critical clathrin polymerization concentration (*11*), and *a* ~ 15 *k*_B_*T* was estimated from a micropipette experiment (*33*). These estimates of *a* appear reasonable in light of the extensive contact area involved in clathrin interactions in lattices (*6*, *9*). We used *a* ≈ 30 *k*_B_*T* for all calculations described in the main text but also considered how changes in the value of *a* modify the CCP energy landscape (figs. S3A and S4A).

Combining Eqs. 1 to 4 and 6, we obtain the total energy of a CCP (Fig. 3E)

$$E_{\text{CCP}}=\frac{A\kappa_{\text{memb}}}{2}{\left(\frac{2}{R}\right)}^{2}+\gamma \frac{A^{2}}{4\pi R^{2}}+\frac{A\kappa_{\text{clath}}}{2}{\left(\frac{2}{R}-\frac{2}{R_{\text{clath}}}\right)}^{2}-a\frac{A}{A_{\text{clath}}}$$ (7)

In contrast to the constant area and constant curvature models of CME (fig. S1, A and B), the CCP energy landscape implied by the above expression for *E*_CCP_ (Fig. 3E) allows a large variety of CCP shapes and maturation pathways, including the occurrence of nonterminal events (note S4) (*14*). The predicted CCP energy landscape suggests that, provided all relevant contributions to the CCP energy are considered in Eq. 7, high-energy CCP states (indicated in red in Fig. 3E) should be sparsely populated, while lower-energy CCP states (indicated in yellow and green in Fig. 3E) should be more densely populated. Furthermore, Eq. 7 predicts toward what CCP morphologies CCP maturation can convene spontaneously without any energy input, i.e., the low-energy area indicated in blue in Fig. 3E and, more precisely, the lowest-energy CCP state with *A ≤* 4π*R*^2^ (indicated by a red circle in Fig. 3E). The basic morphology of the predicted CCP energy landscape is robust with respect to a wide range of perturbations to the values of the parameters in Eq. 7 (figs. S3 and S4). The generic predictions of Eq. 7 are in good agreement with the measured CCP probability map (Fig. 3F). For the values of κ_memb_, γ, κ_clath_, *R*_clath_, and *a* used here, Eq. 7 predicts that the lowest-energy CCP state takes the shape of a sphere with radius *R* ~ 55 nm (note S4). This optimal CCP shape emerges from the competition between *E*_clath-bend_ that favors CCPs with *R*_clath_ ≈ 40 nm (Fig. 3C), *E*_clath-poly_ that favors growth of CCP area (Fig. 3D), and *E*_memb-bend_ and *E*_memb-tension_ that favor flat CCPs (Fig. 3, A and B). The spherical CCP radius predicted by Eq. 7 matches well with the observed radius *R* ~ 55 nm of CCVs formed by PTK2 cells (*24*, *25*).

How do the two traditional CCP maturation models, the constant radius and the constant area models of CME, connect to our CCP energy landscape? The constant radius model (fig. S1B and note S4) is well situated in the energy landscape: the minimum energy path of CCP formation is approaching this model at low membrane tension values (Fig. 3E and fig. S1C, path 1). In contrast, the initial stages of the constant area model (fig. S1A), i.e., the observed low-curvature clathrin lattices (*7*, *42*, *43*), are located in highly unfavorable regions of the predicted CCP energy landscape (Fig. 3E). According to Eq. 4, tens of *k*_B_*T* of bending energy are stored in individual clathrin interactions in approximately flat CCPs (Fig. 3G). Thus, in such lattices, the energy gain due to clathrin polymerization is expected to be comparable in magnitude to the energy cost of clathrin bending, making low-curvature clathrin lattices prone to local dissociation (perhaps aided by other components in the cellular context; see Discussion). Thus, the CCP energy landscape predicted by Eq. 7 suggests that the clathrin bending energy cost associated with flat CCPs facilitates maturation of such CCPs toward more highly curved CCP states, producing “jumps” in the CCP energy landscape as clathrin arm dissociation releases architectural constraints in (flat) clathrin lattices and, in turn, allows renewed clathrin arm polymerization in energetically more favorable (more curved) configurations.

### Flat clathrin lattices are elastically loaded networks

The above theoretical predictions qualitatively match HS-AFM observations where multiple CCPs emerged from what seemed to be a larger clathrin lattice that broke into smaller and more curved CCPs (Fig. 1A, middle left, and fig. S5), and an amassment of platinum replica EM observations (*7*, *42*). Furthermore, AFM provides the unique ability to mechanically manipulate the sample (*16*, *44*, *45*), which allows direct tests of our theoretical predictions. We used the HS-AFM tip as a nanoscalpel to dissect clathrin arm interactions. Inducing local breakages in CCPs should lead—if our theoretical rationale was correct—to a local increase in CCP curvature and reshaping of the CCPs according to the CCP energy landscape.

**Fig. 4:**
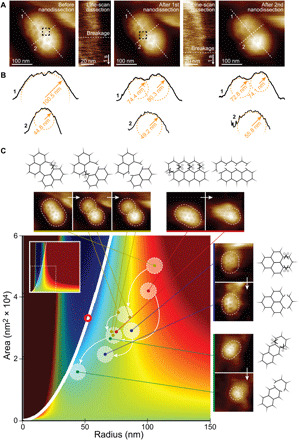
HS-AFM nanodissection: Low-curvature clathrin lattices are loaded with elastic energy. (**A**) Details of the clathrin lattice nanodissection experiments. A clathrin lattice on a plasma membrane before (left), after the first (middle), and after the second (right) nanodissection, with the corresponding targets for nanodissection (dashed squares) and single line scans over individual clathrin arms at gradually increased applied force to dissect individual clathrin-clathrin arm-arm interactions (insets). (**B**) Section analysis along the white dashed lines “1” and “2” in (A): The lattice is initially rather flat along line 1. Following the first and second nanodissections, the section analyses show the lattice separating into two separate CCPs with increased curvature (top profiles 1). In contrast, the nanodissected region is pulled down and flattened (bottom profiles 2). (**C**) Clathrin nanodissections lead to release of clathrin lattice constraints, allowing low-curvature CCPs to converge to energetically more favorable high-curvature CCP states (CCP energy landscape reproduced from Fig. 3E). As in Fig. 3E, the red bold circle indicates the lowest-energy CCP state with *A ≤* 4π*R*^2^, which lies on the *A =* 4π*R*^2^ curve.

We first identified clathrin arms that maintained architectural constraints in low-curvature CCPs, notably clathrin-clathrin interactions in extended hexagonal regions in CCP HS-AFM images (Fig. 4, A and C). We then targeted individual clathrin arms one after another and performed nanodissection by applying increased force. After each nanodissection, images of the resulting CCPs were taken. As predicted by the CCP energy landscape, we found that CCPs broke into smaller CCPs with decreased radius of curvature (Fig. 4A). Notably, while we observed the newly formed CCPs to bend spontaneously to smaller radii of curvature (Fig. 4B, profiles 1), the curvature in between these CCPs decreased (Fig. 4B, profiles 2). The spontaneous emergence, following mechanical perturbation, of two CCPs with increased curvature should be energetically unfavorable, would there not be elastic energy stored in the clathrin lattice.

Various nanodissection experiments of low-curvature clathrin lattices (Fig. 4C, lattice schematics) led to different clathrin lattice deformations (Fig. 4C, HS-AFM images) but always caused curvature increases when lattice constraints were released (Fig. 4C). Tracking the evolution of the nanodissected clathrin lattices on the combined CCP energy landscape, we found that the newly isolated CCPs adopted energetically more favorable states, producing jumps in the CCP energy landscape tending toward energetically more favorable CCP morphologies (Fig. 4C, main panel). Together with our predicted CCP energy landscape, the above observations suggest that clathrin bending deformations elastically load low-curvature CCPs, priming CCPs for their maturation toward highly curved CCVs.

## DISCUSSION

Here, we propose a theoretical framework and provide experimental evidence suggesting that clathrin coats act as elastically loaded networks. Of course, CCPs are extremely complex systems, and correlative microscopy approaches reported that up to 18 proteins can be found together with clathrin at various CME instances in CCPs, some of which have been shown to participate in CCP topological maturation (*46*, *47*). Therefore, “clathrin coats” can be understood throughout this work as the ensemble of components that drive CME, with clathrin playing a central role in setting the mechanical properties of CCPs. Because of the intrinsic molecular curvature of clathrin triskelia, low-curvature clathrin coats are elastically loaded. Inducing, without compositional alterations, local breakages in CCPs through nanodissection, we found a pronounced increase in CCP curvature, which shows that the clathrin network plays a major role in CCP mechanics. Further in-depth studies will be necessary to pinpoint the contribution to and modulation of this mechanism by other molecules involved in CME.

A recent cryo-EM study of self-assembled clathrin cages provides interesting complementary insights (*9*). In equilibrium conditions, purified clathrin forms small cages with a narrow size distribution comprising between 28 and 36 triskelia (table S1) (*9*), with the triskelia engaging in polygons of type 6-6-5, 6-5-5, and 5-5-5. The highly curved 6-5-5 triskelion was found, by far, to be most abundant and is therefore expected to be closest to the energetically preferred curvature of clathrin. The trimerization area of all triskelia in all cages was found to be highly curved and roughly threefold symmetric independent of the type of adjacent polygon (*9*). Forcing such triskelia into an approximately planar geometry in extended hexagonal lattices is expected to be highly unfavorable from an energetic perspective.

The elastically loaded lattice model of CCP maturation presented in this work yields an energy landscape that allows classification of the observed CCP shapes and encompasses the previously proposed constant area and constant curvature models of CME (Fig. 3E). Hence, this model not only incorporates all observations that have previously been explained by these two models but, crucially, also allows CCP shapes that cannot be fully explained by either model. The CCP states that, according to the constant radius model of CME, should lie along the CCP maturation pathway are among the most favorable CCP states in the CCP energy landscape (note S4). Nevertheless, many CCPs in unroofed cells are quasi-flat or have a radius of curvature that is incompatible with continuous growth of the CCPs at constant radius toward a realistically sized CCV, as implied by the constant curvature model of CME. The CCP energy landscape suggests a different maturation pathway for such lattices. Our model indicates that the architectural constraints imposed on clathrin triskelia in flat lattices give rise to intrinsic bending frustration in flat CCPs (*7*, *23*). The energy penalty associated with this geometrical frustration only depends on how much the CCP radius of curvature deviates from the intrinsically preferred radius of curvature of clathrin and is independent of the lattice size. However, in a larger lattice, many more clathrins are stressed and, thus, larger flat lattices are rarer and more likely to break. The resulting newly isolated CCPs, where triskelia are released of their lattice constraints, are predicted to adopt higher curvature states that conform better to the intrinsic triskelia curvature, followed by growth through polymerization of potentially unconstrained triskelia. This sequence can be repeated to yield CCVs (fig. S1C). One may view this CME pathway as a series of CCP growth spurs at constant radius, interspersed by lattice breakages that bring the CCPs closer to the final CCV radius.

To experimentally demonstrate that low-curvature CCPs are elastically loaded, we used HS-AFM nanodissection to induce lattice breakages that were spontaneously followed by curvature increase. In cells, many factors both exterior [e.g., cargo loading (*48*)] and interior [e.g., forces exerted by actin (*49*), feeble uncoatase action (*10*), or association of other membrane bending–prone molecules such as dynamin, F-BAR proteins, or epsin] might shift the energy balance toward higher curvature CCP states (*50*). We thus propose that extended, flat, and elastically loaded clathrin lattices could represent ready-to-use CME platforms that, upon outside cargo binding or interaction with cytoplasmic components, can swiftly and efficiently be activated, akin to the firing of a projectile through release of a loaded spring.

The elastically loaded lattice model of CCP maturation reconciles to a large extent the earlier constant area and constant curvature models of CME and may explain a range of previous observations: (i) Successful CME events may evolve from the recurrently observed large flat clathrin lattices (*7*), (ii) clathrin turnover reported in Fluorescence Recovery After Photobleaching (FRAP) experiments (*13*, *51*, *52*) may represent optical signatures of breakage and growth cycles necessary to evolve large flat lattices, (iii) the energy required to break flat clathrin lattices (*33*) may be reduced by the large elastic energy penalty imposed on flat clathrin-clathrin interactions, (iv) the probability of breakage increases with lattice size and provides a size control mechanism (*53*), (v) the CCP energy landscape model predicts the occurrence of nonterminal failed endocytic events (*14*), and (vi) the decrease/inhibition of CME in cells under high membrane tension such as during mitosis (*54*) or actin inhibition (*55*). The calculated CCP energy landscape predicts that, under high enough membrane tension (>0.06 *k*_B_*T*/nm^2^ for the parameter values used here), the CCP energy minimum may lie to the right of the *A =* 4π*R*^2^ curve, which means that formation of spherical CCVs becomes energetically unfavorable and CME should stall under such conditions (*33*). Finally, (vii) the recurrent observations of highly matured CCPs next to large, flat clathrin lattices likely represent snapshots of evolving CCPs after breakage from such elastically loaded clathrin lattices (*7*, *42*, *43*, *56*, *57*).

Together, we propose a model for CCP maturation that is nonprocessive but described by a CCP energy landscape (Fig. 3E and fig. S1C). In this model, the driving force for CCP maturation is clathrin polymerization that, combined with the intrinsic curvature of clathrin triskelia, yields storage of rapidly accessible elastic energy in flat clathrin lattices. A similar concept has recently been proposed for the ESCRT-III system that is also involved in membrane deformation (*18*). Furthermore, a similar release of elastic energy has been put forward to explain the speed of synaptic vesicle fusion (*58*, *59*). Physical cues targeting release of stored elastic energy might provide a common mechanism in protein action—especially in processes that require a speedy supply of large amounts of energy such as in membrane deformation, fusion, and fission processes. A lack of suitable experimental approaches in the cell biology and biochemistry toolboxes has so far hindered quantification of these physical cues. Optical tweezers, HS-AFM, and other high-precision experimental techniques are now helping to elucidate the role of elastic loading in protein reorganization.

## MATERIALS AND METHODS

### Cell unroofing

PTK2 cells are cultured at 37°C in 5% CO_2_ in Dulbecco’s modified Eagle’s medium (Gibco, USA) with 10% (v/v) fetal bovine serum (Gibco, USA). The PTK2 cells are particularly suited for unroofing because of their large (>100 μm) size, flatness, and Clathrin Light Chain - Green Fluorescent Protein (CLC-GFP) expression. To prepare unroofed cells, PTK2 cells are grown overnight until confluence on AFM glass cylinders that have been incubated beforehand in 0.01% poly-d-lysine (Sigma-Aldrich, USA) for 15 min and rinsed with 1× phosphate-buffered saline (PBS) three times (Sigma-Aldrich, USA). For the following manipulations, the glass cylinders with grown cells are placed into a 30-ml beaker and fixed on the bottom of the beaker with silicon grease. The cells are rinsed several times: one time with 37°C PBS solution to remove cell culture medium, followed by three times rinsing with buffer A kept on ice (30 mM Hepes, 70 mM KCl, 5 mM MgCl_2_, and 3 mM EGTA, adjusted with KOH to pH 7.2, threefold diluted with deionized water). After rinsing, the cells are “unroofed” in buffer A by sonication on ice using an Osonica Q500 probe sonicator (Qsonica, USA). The sonication parameters are as follows: distance between the sample and the probe, 1 cm; probe diameter, 1/8″; amplitude, 20%; and sonication time, 1 s. The unroofed cells are stained with FM 1-43 FX (Life Technologies, USA) membrane stain diluted to 5 μg/ml in buffer A. Cells with fluorescently labeled lipid bilayers are briefly tested for successful unroofing in a fluorescence microscope before HS-AFM imaging—successful unroofing is characterized by the presence of extended plasma membrane sheets located in the focal plane near the substrate surface only.

### HS-AFM imaging

All AFM images were acquired with an amplitude modulation mode HS-AFM [Research Institute of Biomolecule Metrology (RIBM), Japan] using short cantilevers (USC-F1.2-k0.6, NanoWorld, Switzerland) with spring constant of 0.6 N/m and resonance frequency of ~0.6 MHz in buffer. The scanning speed was 3 s per frame for 300 pixel × 300 pixel images using optimized feedback operation (*16*).

### HS-AFM nanodissection

Each clathrin arm interaction targeted for nanodissection was selected during HS-AFM imaging. The AFM tip was positioned above individual clathrin arms through imaging at very small scale. Subsequently, line-scan imaging perpendicular to the arm was performed at gradually increased applied force, i.e., decreased ratio (down to *A*_set_/*A*_free_ ~ 0.5), where *A*_set_ is the set point amplitude and *A*_free_ is the free oscillation amplitude of ~2 nm (*45*), reaching up to ~500-pN loading force. Line-scan imaging was performed until the selected arm was broken. Subsequently, *A*_set_ was reset to *A*_set_/*A*_free_ ~ 0.9 and the HS-AFM was set to large-scale imaging mode.

### Image processing

All HS-AFM images were drift-corrected using StackReg in ImageJ (*60*) and adjusted by self-written image analysis software in MatLab (MathWorks, Natick, MA, USA). Sphere fitting was performed by fitting the parametrized sphere equation into the HS-AFM topographic data using a linear least squares–based MatLab fitting function (*61*). To avoid sphere fitting errors due to tip dilation of the topography, only the data points on the top ±45° of the CCP topography were fitted. Clathrin lattice detection followed by lattice spatial analysis was carried out using self-written routines in MatLab. In brief, topography images are duplicated and one of the images is high pass–filtered to only retain the high frequencies, i.e., the clathrin lattice, while the overall topography is filtered out. Next, peak detection is performed where pixels that are surrounded on two sides by lower pixels are selected and set to 1 in a binary image. This step is performed three times, once on the original image (sampling 1) and twice on down-sampled images (samplings: 0.5 and 0.25), to increase the probability to detect the top of flat peaks. Several rounds of pixel dilation and erosion were performed to correct for missed discontinuous detections.

### Clathrin purification and clathrin cage polymerization

CCVs were purified from fresh calf brain using differential centrifugation as described by Campbell *et al.* (*62*). Subsequently, clathrin was dissociated from the membrane vesicles in a buffer containing 0.5 M tris-HCl (pH 7.0), 1 mM EDTA, 0.2 mM dithiothreitol (DTT), 0.02% (w/v) sodium azide, and phenylmethylsulfonyl fluoride (PMSF; 50 mg/liter; freshly added) and polymerized into cages by 10-fold dilution in a polymerization buffer with 10 mM MES-NaOH (pH 6.5), 100 mM KCl, 1 mM EGTA, 0.5 mM MgCl_2_, 0.2 mM DTT, 0.02% (w/v) sodium azide, and PMSF (50 mg/liter).

### Elasticity measurements

Two microliters of a clathrin cage sample was incubated on freshly cleaved mica for 1 min at room temperature. HS-AFM (RIBM, Japan) imaging was used to target individual clathrin cages. The tip was placed on top of each cage, upon which force curves were acquired using short cantilevers (USC-F1.2-k0.6, NanoWorld, Switzerland) with a nominal spring constant of 0.6 N/m and a resonance frequency of ~0.6 MHz. All elasticity measurements were performed in polymerization buffer.

## SUPPLEMENTARY MATERIALS

Supplementary material for this article is available at http://advances.sciencemag.org/cgi/content/full/7/33/eabg9934/DC1
